# The Selection and Training of Shelter Dogs for Involvement in Canine-Assisted Interventions: What Are the Ethical Issues?

**DOI:** 10.3390/vetsci12050497

**Published:** 2025-05-19

**Authors:** Laura Contalbrigo, Elizabeth A. Walsh, Lieve L. Meers, Daniele Benedetti, Marta De Santis, Emma Bassan, Simona Normando

**Affiliations:** 1 National Reference Centre for Animal Assisted Interventions, Viale Dell’Università 10, 35020 Legnaro, PD, Italy; lcontalbrigo@izsvenezie.it (L.C.); mdesantis@izsvenezie.it (M.D.S.); ebassan@izsvenezie.it (E.B.); 2 Cork Pet Behaviour Centre, Clonakilty, P85 YF58 Cork, Ireland; 3 BIAAT Foundation, Stadsplein 100/12, 3600 Genk, Belgium; info@biaat.be; 4 Local Health Unit Rome 6, Via Borgo Garibaldi 12, 00041 Albano Laziale, RM, Italy; daniele.benedetti@aslroma6.it; 5 Dipartmento Di Biomedicina Comparata E Alimentazione, Agripolis, University of Padua, Viale dell’Università m16, 35020 Legnaro, PD, Italy; simona.normando@unipd.it

**Keywords:** ethical matrix, shelter veterinarians, canine-assisted intervention, selection and training in CAI, ethical guidelines for CAI, behavioral assessment, health risk

## Abstract

Managing relinquished/stray dogs is a significant socio-economic and ethical challenge necessitating structured policies to ensure animal welfare and integration into society. Critical to successful rehoming is careful assessment of a dog’s temperament/behavior and appropriate matching of a dog to an owner. Shelter dogs’ involvement in canine-assisted interventions (CAIs) provides a potential avenue for rehoming through appropriate dog educational programs. CAIs consist of structured interactions between dog and clients targeted at specific groups with physical/psychological/emotional impairments in various settings. However, there is a lack of standardized protocols for assessing shelter dogs’ suitability for CAI, raising ethical concerns regarding selection/training practices. This study explores the ethical dimensions of rehoming shelter dogs for CAI, employing a philosophical framework considering the responsibilities of humans/veterinarians toward companion animals and how societal perceptions of human–animal relationships impact decision-making in this context. An ethical matrix is applied to evaluate the implications of rehoming decisions on canine and human interests. Our findings highlight the need for a balanced, responsible approach that respects the individuality of dogs while promoting their potential roles in human society. Ethical guidelines for the selection and training of shelter dogs for CAI could enhance their welfare, improve adoption rates, and reinforce the value of the human–animal bond.

## Simple Summary

Dogs who are strays or surrendered to shelters pose a financial and ethical issue regarding ensuring their welfare and future. It is important to assess each dog for temperament and health to enable matching with a suitable owner. Canine-assisted interventions (CAIs) may provide a solution for dogs that is potentially suitable for this. However, currently, procedures do not exist for assessing shelter dogs for CAIs. This causes ethical questions regarding how these dogs might be selected and trained, and the ethics applied. This paper looks at the ethics of rehoming shelter dogs for CAIs, using a philosophical approach to consider human/veterinary responsibility for animals, and how beliefs regarding human–animal interactions influence the outcome, using an ethical matrix to evaluate the effects of rehoming shelter dogs. This paper draws attention to the need for an approach which is balanced and responsible with respect to each individual dog, and which emphasizes possible roles for them in society. Shelter dog welfare can be improved by having ethical guidelines for their assessment and training, which can increase adoption rates in shelters and promote the human–dog bond.

## 1. Introduction

Relinquished or abandoned dogs and stray dog population management are social and economic issues that affect the public veterinary system and animal welfare organizations [1]. In Europe, there are approximately 100,000,000 stray dogs, and the adoption of political strategies at EU level is the most viable approach for managing the canine population, promoting the responsible care of companion animals, and bringing this situation under control [2]. Unfortunately, it is not currently possible to present a figure for dogs housed in European shelters, due to many factors, including the non-uniformity of reporting within countries, the fluidity of dogs entering and leaving shelters, and the wide variance in welfare legislation within EU member states [3]. These may be stray dogs or the dogs may have had owners who would not or could not take care of them any longer, perhaps due to factors such as the health or behavior of the dog or to a change in the owner’s lifestyle. They may be returned to the shelter because the rehoming process fails [4] or they may be dogs rescued from conditions of animal abuse [5,6,7,8]. It is incumbent on and incorporated into the employment description of the veterinarian responsible for the shelter to bear responsibility for all decisions relating to the veterinary assessment and treatment of all dogs, including physiological and behavioral assessments, decisions regarding the dog’s suitability for adoption, and the ultimate decision of euthanasia (dependent on legislation within that country; some countries such as Italy have a “no-kill” policy) [9,10].

A critical issue for all shelters is a successful rehoming rate for dogs that is based on an effective assessment of the animal and a good matching process with the potential new owner [11]. Reintroduction of shelter dogs in human society is a challenge, especially for long-term shelter dogs that seem to be the most affected, possibly exhibiting behavioral and stress-related problems with detrimental effects on their relational skills [12]. Therefore, re-education programs coordinated by behaviorists with the support of adequately trained staff are required before the rehoming of these dogs both as companion animals and as working dogs for social utility. Shelter dogs who might be suitable for canine-assisted interventions (CAIs) could act as ambassadors to improve opportunities for all shelter dogs, impacting positively on the image of shelter dog ownership and on the whole of society, with the appropriate dynamic structure, competent behavioral investment, and careful, balanced matching of owner and dog.

Indeed, one of the human beneficial services that mainly involves dogs (which may be termed “therapy dogs”) is CAI dogs, as a consequence of a dog’s natural aptitude for relating to humans, based on their long history of co-evolution with the human species [13,14,15].

CAIs consist of positive structured interactions between humans and dogs, aimed at improving the health and wellbeing of the patients/clients involved. They can be targeted at people with various physical, psychological, or emotional issues, in various settings [16]. In CAIs, all aspects of the health, safety, and welfare of both the people and animals involved must be considered, in order to conduct ethically sound and successful interventions [17,18,19,20]. With regard to the canine candidates for CAI, veterinary involvement in the roles of both animal health professionals and advocates of animal welfare is crucial [21]. Importantly, the involvement of dogs in CAI requires a thorough veterinary assessment of their health and behavior, with due attention to the individual anamnesis [22,23,24].

Currently, there are few clear indications or protocols for the selection of eligible shelter dogs for CAI and therapy dog training programs [25,26]. There is also a lack of standardized operational working methods and clear requirements [27], according to which the recovery of social and intra- and interspecific relational skills of shelter dogs could be considered sufficient to be successfully involved in CAI. The shelter dog training program should be developed to address the specific needs of each dog, and it can include sessions to improve social and intra- and interspecific relational skills, in addition to some basic obedience if needed. All training should be reward-based.

On the other hand, the situation of animal shelters raises some concerns, particularly as regards the animals’ quality of life and the services aimed at providing opportunities for their adoption. Therefore, a comprehensive ethical analysis of the selection and rehoming practices of shelter dogs to be involved in CAI seems to be appropriate, considering all the relevant values linked to this issue. Decisions about one dog may have consequences for other dogs and human welfare, impacting on other human values or on the environment, and the manner in which people perceive and evaluate all these issues affects their ethical decision making.

The aim of this paper is to discuss ethical issues involved in the selection of shelter dogs showing potential to be considered as possible CAI dogs.

In this paper, a broader conceptual framework concerning the relationship between humans and companion animals, especially dogs, will be considered, investigating the interests of CAI dogs, shelter dogs, and humans from the perspective of CAI [28]. Finally, all aspects and interests associated with the selection and training of shelter dogs for involvement in CAI will be analyzed and included in an ethical matrix based on the one introduced for the first time by Mephan et al. [29].

## 2. Ethics in the Human Companion–Animal Relationship

Since the duty to take care of the health and wellbeing of domestic animals rests on the human-dependent relationship they share with us, this relational perspective is at the core of a cluster of theories which can be called “relational theories”, in which the type of human–animal relationship is crucial to the definition of their right treatment. The “second wave” of the feminist movement connected the so called “feminist theories” with ecology and animal rights [30]. Feminist theories focus on sympathy, empathy, and compassion as essential elements of the human–animal relationship, calling for an effort to communicate cognitively and emotionally with animals [31,32,33].

In contrast with the Western, mathematical, male psychology, which results in the domination of nature, and hence of animals and women [34], the feminist perspective presents a relational, affective approach. Feminist theorists focus on the differences and varieties among the species, rather than the similarities, but not in the direction of a hierarchical, manipulative, and exploitative mode of relation.

Such a relationship should be characterized by a caring or holding attitude; this is typically a mother’s attitude, focused on taking care of the other and protecting the fragile. Therefore, this “maternal ethic” [35] represents a “morality of responsibility”, as opposed to the “morality of rights” supported by the male, formal mode of thinking [36].

There are two basic conditions for taking this responsibility. The first one is to consider the animal as an “other” that has its own value and dignity, and, as in the case of companion animals, can even be an “active subjectivity” in the relationship. Therefore, the animal cannot be conceived merely as an instrument or as a thing (reification), nor can it be compared to humans for its features and needs (anthropomorphism). The second condition is to adopt an attitude of attentiveness and availability towards that “otherness”, that is, to be open to their reality, experiences, and needs. This in turn makes it possible to be preoccupied with their wellbeing and hence to take care of it.

This attitude of openness towards the “other” also reveals a radical asymmetry between humans and animals in terms of strength and power which does not lead to domination and exploitation, but rather to augmented responsibility.

Moreover, the asymmetry implies the absence of reciprocity, suggesting that humans have responsibility to care for the health and wellbeing of animals without expecting anything in return. Indeed, other researchers such as Palmer [37] highlighted that the anthropo-dependence developed by domestic animals results in a surplus of human responsibility for them, in terms of providing for their needs and protecting their interests. This analysis still refers to the “pet-ownership” culture, where the human–companion animal bond emphasizes animal protection needs and a tendency towards anthropomorphism.

Nevertheless, since early 2000, while maintaining the same ethical framework there has been a cultural shift towards the “pet-partnership” vision of the human–companion animal bond. This is a collaborative kind of relationship in which companion animals and humans work together in social activities useful for human communities [38,39]. The effect of this cultural shift is a change in the integration of the pet (companion animal) in human society which moves towards the adoption of a new perspective.

Currently, a companion animal represents as an “other” with a peculiar relationship with society and individual citizens, which is different from the human-to-human relationship in many and varied environments, both natural and those designed and built by humans for humans. Adopting a zoo-anthropological approach, the presence of companion animals, especially dogs, in this human context can be considered an additional social value for citizens both in the “individualized relationship” and in the “assistive and educational relationship” expressed through animal-assisted interventions. Therefore, a new moral duty arises for human society, creating the conditions for the various expressions of such a bond in public contexts for both companion animals and human-interest satisfaction.

## 3. Animal Interests: CAI Dogs and Shelter Dogs

The very concept of “animal interest” allows for an interdisciplinary approach, since it lends itself to containing both scientific notions and ethical considerations. In general, the term “interest” is used to indicate that which is beneficial for someone. More specifically, Roberto Marchesini links the notion of interest to other terms like suffering, welfare, health, preference, wellbeing, telos (purpose), and zoo-anthropological wellbeing [38].

Animal health and welfare concepts are closely linked, including not only the absence of suffering, but objective animal-based measures of physiological, ethological, and psychological parameters [40,41], which provide information about the state of the animal’s body and mind. These include self-perception, feelings, and emotions, which assist in defining animal wellbeing and include not only the expression of innate preferences, based on ethological features, but also the complete realization of the individual’s psychological subjectivity [42,43]. Animal subjectivity encompasses innate individual features, (genetics) epigenetic effects, setting up an equilibrium with the environment inclusive of the companion animal, and the human–animal relationship [44].

Zoo-anthropologic wellbeing encompasses all the strategies for a responsible partnership between humans and animals, which have a common interest in what can be called the “pact of domestication” [45,46,47]. This pact is mutually beneficial: humans can gain company, food, and support in various types of work, while animals can gain housing, food, protection, and positive interspecific interactions.

This applies to dogs, in particular, whose domestication history began at least 15.000 years ago [48]. They are entities that cannot exist outside human cultural context [49], where they build a two-way interaction with humans based on trust [50]. Indeed, researchers have considered the human–dog bond based on attachment theory [51,52,53], since an interactive type of relationship develops between dogs and humans in which there is a mutual recognition and a strong emotional connection. In this relationship, dogs can find self-realization and social referencing if the dog and human interests are shared while respecting their different roles [38,46].

This is especially true for dogs involved in CAI, where human responsibility towards dogs should address three moral issues: (a) the assessment of the actual benefits and risks for human health and safety; (b) the duty to ensure the welfare of dogs in all phases of CAI projects from both the physical and psychological points of view; and (c) the commitment to creating favorable conditions for the benefit of both humans and animals involved in the interventions.

In general, there is still a “void in the literature regarding the impact of these interventions on the therapy animals themselves” [54], although there is growing awareness that animal welfare should be considered carefully, and the number of studies on the topic is increasing steadily [55]. Those animals considered as “pets” do not appear to attract the same amount of investigation as farm and wild/captive animals [56].

The major issues of interest to researchers concern animal suitability for the different types of AAI, the animal selection process and training methods, the stress levels which may be experienced by the animal during AAI sessions, the settings’ conditions, and associated risks. Additionally, the aging and retirement of the animals [57,58,59,60,61,62,63,64], the impact of the housing conditions in shelters [65], the transportation of dogs from the kennels to CAI training areas, the effects of previous animal abuse and neglect on the suitability of dogs for CAIs, and the minimum time period a handler and CAI dog should be a team (six months in some CAI programs) prior to taking part in CAIs [66] are all of primary concern. Other issues have received little investigation, such as the functionality of dog–handler matching, and how this can affect dog welfare and the efficacy of the intervention [67,68].

Therefore, the interest of the dog should be included in this process even if it is impossible for us to entirely adopt the animal perspective. However, “any ethical model must be able to at least include the other in its existential horizon” [69]. The interests and quality of life of dogs involved in CAI should be safeguarded by veterinarians through clinical and behavioral examinations that consider the attitude, motivation, and emotion of dogs. Veterinarians should develop procedures to ensure adequate play and rest time for the dog according to his/her work load and resilience [19]. Their welfare needs to be constantly monitored by the dog handler who is the human partner in the CAI setting, using tools that allow an objective measure of both positive and negative effects on the dog [19,70,71,72]. Moreover, the CAI setting needs to be built according to the requirements of the dog, giving him/her a safe place where the animal can retreat in the event he/she needs to avoid interaction with the client. The opportunity to exert agency in involvement in the CAI session or not is key to ensuring dog welfare [19,22]. In conclusion, safe and beneficial dog involvement in CAI evolves into beneficial effects for people, improving the entire CAI program [59].

When we evaluate the possibility of involving shelter dogs that were recently abandoned and/or abused in CAI, the concepts described above need careful consideration and application. Indeed, in the case of dogs housed in shelters, we need to reduce the negative impact of the shelter on dogs’ relational skills, ensuring, as much as possible, appropriate intraspecific and interspecific interactions [65,73] and effective rehabilitation programs targeted at abused animals, for whom a confident relationship with humans has been compromised. At the same time, effective strategies to favor the rehoming of the animal need to be put in place, enhancing dog welfare and reducing dog population and shelter management issues.

Therefore, defining protocols for the selection and training of shelter dogs for their involvement in CAI should be fostered, while being cautious and examining all risk factors [26]. Additionally, varied roles in varied settings and contexts exist for CAI dogs at international level, such as the following:

(1) Training shelter dogs as CAI dogs: shelter dogs are trained by professional handlers, only working with clients in CAI after training and post-adoption.
(2) *Programs in which clients train shelter dogs to be rehomed as pets; the training is part of the CAI program* [74].
(3) *Programs in which clients train shelter dogs to be rehomed as service/assistance dogs; the training is part of the CAI program.*
(4) *Canine-assisted education (CAE) in school programs (shelter dogs are trained by clients as part of a CAI program and post-training they work in CAI/CAA/CAE programs).*
(5) *Programs in which the client is involved in the rescue of the shelter dog* [75].
(6) *Programs where the client trains the shelter dog to accept medical procedures.*

It is noteworthy that the impact on veterinarians and on other stakeholders may vary due to the framework of shelter dogs’ involvement in CAIs, dependent on where and in what format the intervention takes place.

Therefore, this paper is focused on the settings and context as indicated in role (1), in which a shelter dog is assessed as being suitable to be involved in CAI after adoption, and the adopter intends to involve the dog in CAI. Such potential CAI dogs will be chosen to partake in programs and training that nurture and develop their predisposition and suitability to be CAI dogs, and to be placed for adoption as a CAI dog. The programs in italics are not the focus of this study.

## 4. Shelter Dog Interests: Ethics Regarding Portrayal and Perception

The myriad reasons for relinquishment of dogs to shelters are poorly understood [6,7,8] and may include two distinct categories: (i) dog issue-related factors, such as dog behavior [8], health issues, and origin of the dog; and (ii) factors unrelated to the dog, such as training invested and success achieved, owner moving home [5], duration/experience of dog ownership, age of the dog, number of dogs owned, morphology/size of the dog [6], characteristics of the owner [76], owner knowledge level, owner health issues/marital breakdown/death [77], and too-high investment level of effort/work/time in/for the dog [21,78]. The salient point is that the second group of factors relates to dogs who may have been relinquished for reasons unrelated to any direct dog-associated issue, or are not necessarily indicative of a failed or unsuccessful bond, but which may have an impact on a successful rehoming. Neuter status may affect a dog’s temperament positively or negatively, dependent on many factors. In their study, Marston et al. [78] found that accommodation provided the greatest issue for relinquishment for three dog shelters for which archival data was accessed for one year. Mondelli et al. [4] found that in a six-year period, 40% of dogs who were returned to an Italian shelter were returned for unrelated factors or “management reasons”. Additionally, these authors draw attention to the Regional Shelter Survey [6,77], which stresses the deficiency of owner understanding and comprehension in canine husbandry and behavior, causing (presumably) a lack of differentiation between normal dog behavior and problem dog behavior [79]. The perception of problematic behavior in dogs may be dependent on the owner and how they may consider that behavior to reflect on them personally [80]. However, some studies have focused on behavioral problems or “misbehaviours” [81], without adequate consideration of the issues and whether they related to normal canine behavior or to abnormal/unexpected behavior. These factors have created a portrayal of shelter dogs as canines predominantly of poor behavioral character, whereas this may be inaccurate. The accurate portrayal of shelter dogs, through selection and training for CAI, may have the effect of focusing public perception on normal and expected behavior in dogs, as opposed to possibly misinterpreting behavior and perceiving it as problematic, thus giving an opportunity for rehoming to many shelter dogs worldwide.

## 5. Human Interest in Canine-Assisted Interventions

Human interest is a wide concept that sociologists and economists consider a driving force for all human actions or at least a major force that influences human life in association with other factors [82]. Authors agree that an undifferentiated concept of interest is useless and they recognize the need for classification using different types and subtypes. As an example, Small [83] divided human interest into six basic types: health interest, wealth interest, sociability interest, knowledge interest, beauty interest, and rightness interest [83]. In the theories of Coleman [84], interests are linked to two other concepts—”resource” and “control”—developing the idea that, in order to satisfy their interest, an actor needs control over resources that are of interest to them. Moreover, Coleman did not assign interest to a single individual or aggregates of individuals, but he focused his theory on the idea of the corporation or “interest group” [84].

Starting from the thinking of Coleman [84] and addressing the field of CAIs and dog shelters, we can identify some human-interest groups, namely, professionals working in dog shelters (e.g., veterinarians, behaviorists, managers, dog caretakers, dog trainers, dog/animal handlers, volunteers); professionals working in CAI (e.g., veterinarians, behaviorists, dog trainers, therapists, dog/animal handlers, teachers, coaches); clients of CAI; the general public; and public authorities financing animal shelters or CAI, and who develop governance strategies for shelter dog populations or for CAI services.

The abovementioned human-interest groups represent the different perspectives from which the issue must be analyzed from the human perspective. Considering Small’s interest classification, we identify human health and sociability interest at both individual and societal levels as the priorities for CAI patients/clients, whereas wealth, knowledge, and rightness interests could be the primary interests for public authorities and professionals working in dog shelters and in CAI. Indeed, CAIs are services that can produce benefits for people and work for professionals involved. At the same time, implementing the process of shelter dog selection and training for involvement in CAI guarantees a rise in rehoming and a reduction in shelter management issues, and supports rightness interests of dog handlers and veterinarians.

However, it is important to note that the involvement of shelter dogs in CAI could create an increase in the perceived burden of responsibility for the veterinarian. Concerns may arise, especially if potential clients may have immune suppression, which could be compromised by the involvement of shelter dogs in CAI, particularly in relation to infectious diseases [85,86,87]. Even the very best, well-run shelters are examples of anthropogenic biological instability, increasing the risk of transmissible diseases, as they often house transient, displaced, and mixed populations of dogs, many of whom may have received minimal/no prior health care, and/or have a history of being strays, possibly scavenging for survival [88]. For example, *Leptospira* spp. infection (a zoonotic disease) is common among stray dogs in some geographical areas [89], and dogs eliminating the bacterium in urine can be asymptomatic or symptoms can be non-specific [90]. A definitive diagnosis may require additional confirmatory tests for the direct or indirect identification of the pathogen [90], which is unlikely to be affordable for all dogs in a shelter. Therefore, the possibility of placing dogs for adoption as suitable for CAI is likely to cause an increase in resource allocation and expenditure to ensure that the dog is clinically healthy, and this may cause resources required for other veterinary areas within the shelter to be depleted. Such a conflict of resource allocation may create difficult decisions, and potentially a conflict of interest between shelter management (budgeting) and the shelter veterinarian (risk avoidance).

Evaluating the behavior of a dog and their suitability for CAIs in general, and for a specific CAI setting in particular, is a responsibility that veterinarians/behaviorists are likely to have. Usually, the behavioral history of the dog, related by proxy, has an important, but not exclusive, role in such a decision. Access to the behavioral history of shelter dogs, especially for the time before entering the shelter, is likely to be limited, if available. Additionally, temperament testing dogs in the shelter environment could have a limited external validity [91,92], and this may cause the veterinarian stress/distress when deciding to include a shelter dog in CAI. It may also lead to moral conflict, in that shelter dogs assessed as suitable for CAI or trained for CAI may have a potentially higher chance of being considered for adoption. There is a moral responsibility to ensure that dogs with the potential to be CAI dogs are not denied the opportunity, and that those who are selected for CAI pose minimum risk in CAI interactions. The risk of a dog bite to any client is real in a situation where a dog’s issues have not been recognized through either poor display of body language by the dog, or through lack of recognition of it, during assessment [93,94].

However, a key role is played by economic factors, from the perspective of human-interest analysis. Shelter managers and public administrations would benefit from rehoming of shelter dogs to handlers working in CAI, reducing the number of dogs housed and, as a consequence, costs for shelter management. This is particularly the case in countries like Italy, where law [9] forbids euthanasia of shelter dogs with detrimental consequences for facilities that are often overcrowded, in turn impacting the service provided to shelter dogs. Regarding economic advantages for the dog handler, considerations are limited by the lack of economic data regarding CAI dog costs. We can hypothesize that buying a puppy from a breeder and following the specific education and training programs with a dog instructor for approximately two years is more expensive than adopting an adult dog from a shelter with a good prosocial attitude towards humans and motivated towards involvement in CAI. The costs of training adult dogs displaying the ability and having a suitable attitude for CAI are envisaged to be limited from both the economic and time investment perspectives. This is anticipated even if cost-effectiveness analysis research should be required to competently assess the economic gain of this approach.

## 6. Building an Ethical Matrix for Shelter Dog Involvement in CAI

All the abovementioned elements can be included in an ethical matrix, which is a useful tool to help in the ethical analysis of the subject matter of this work. Based on the ethical matrix introduced for the first time by Mepham in 2006 [29], and which was designed to facilitate ethical deliberation in the field of novel biotechnologies, our matrix should help users identify the ethical issues related to the selection and training of shelter dogs for their adoption and involvement in CAI [29].

As in the original matrix, some prima facie principles are applied to a number of interest groups [27,29] already identified, which resulted from the dog and human-interest analysis.

A prima facie principle defines an obligation which is valid in general, but may take second place according to the circumstances. The principles included in the matrix are respect for wellbeing, autonomy, and fairness. They represent the theoretical part according to Beauchamp and Childress [95,96], and are as follows:

(a) Respect for wellbeing representing the utilitarian approach, aiming at maximizing the good;
(b) Respect for autonomy representing the deontological approach, which focuses on the treatment of others not as mere means, but as ends in themselves;
(c) Respect for fairness reflects respect for justice, often related to economic issues such as sustainability.

By “interest groups” we mean all parties concerned, namely, dogs (shelter dogs and CAI dogs); professionals working in shelters (managers, operators, volunteers, veterinarians, behaviorists, and dog trainers); professionals working in CAI (veterinarians, behaviorists, dog handlers, dog trainers, therapists, teachers, and coaches); clients/beneficiaries; people who want to adopt a shelter dog as a pet; public authorities; and society. They represent the different perspectives from which the issue at stake must be analyzed. The ethical matrix is summarized in Table 1.

## 7. Conclusions

Shelter dog population management and CAI issues in which veterinarians can play a pivotal role are two very sensitive fields, as they deal with vulnerable aspects of human society and its often inconsistent relationship with companion animals. Operating in them today implies careful consideration of the consequences of choices that have an ethical impact on people and animals. In our paper, in accordance with our aim, we outline a wide framework for human–companion animal relationships drawing on feminist theories to emphasize a morality of responsibility of humans toward domestic animals and ethics of care. This emerged as an appropriate framework in which we consider the treatment of the “other”, represented by companion animals, as a partner.

Dog and human-interest analysis was performed to identify interest groups and addresses the principal interest types: health, sociability, and rightness for CAI dogs, shelter dogs, and humans, stressing the need for adequate risk assessment in shelter dog selection protocols and training programs to guarantee the health, welfare, and safety of both the humans and dogs involved. This has implications for the best practices for shelters, trainers, and veterinarians. These players’ policy documents should highlight the ethical implications of all interactions between dogs and players. Also, such analysis highlights the importance of implementing shelter practices, such as increased positive dog–person interactions and walks, and a low dog–person ratio, which could increase dogs’ sociability or the likelihood of human socially oriented dogs retaining their social attitudes and increasing their opportunities to be selected as CAI dogs [97,98,99,100].

The veterinarian has an important role regarding both health and safety risk assessment. The perceived increase in the burden of responsibility arising from such assessments with shelter dogs may increase their work-related stress, due to actual/perceived increased health/safety risks, when selecting shelter dogs as candidates for CAI. Additionally, the veterinarian may experience increased distress/compassion fatigue [96] when this perceived burden is evaluated in relation to selecting a shelter dog for CAI, possibly conferring an increased chance of adoption on that dog, in comparison to remaining a shelter, non-CAI trained dog.

Finally, we consider also wealth interests with the economic issues related to the rehoming of shelter dogs and their involvement in CAI. This may be the area in which more research is needed for a broader insight into the economic situation of shelters and for a deeper knowledge of the economic implications for CAI. Our analysis of interests and the use of an ethical matrix allow possible conflicts among the stakeholders to be anticipated, avoiding the polarization of ideas in favor of a common agreement addressing the wellbeing of animals and people, and balancing the perspectives of all the interest groups and the prima facie principles representing ethical theory. Its aim is not to drive a successful “decision making process”, but to set up a method to assess the issue in a pluralist and neutral framework that considers all its facets.

## Figures and Tables

**Table 1:** Ethical matrix for shelter dogs’ involvement in CAI.

Respect for:	WELLBEING	AUTONOMY	FAIRNESS
**SHELTER DOGS to become CAI DOGS**	Welfare, health, wellbeing; safety; improvement in quality of life. Enhanced positive human contact. Enhanced possibility of being adopted, either by a person intending to take part in CAI or as a result of the training/interactions received during CAI.	Behavioral freedom; possibility to exit CAI if appropriate.	Intrinsic value; being recognized as being of the same intrinsic value as non-shelter dogs; same duty of responsibility and care for them. Being given an equal opportunity to find a good home/to live a life worth living. Being able to retain and nurture their predisposition to be CAI dog (if applicable) in the shelter environment. Being recognized as a sentient being.
**Other SHELTER DOGS**	Welfare, health, wellbeing; safety; improvement in quality of life; possibility of reducing competition over shelter resources. Positive consequences of general management/housing procedures within the shelter environment (possibly including enhanced positive human contact) implemented to facilitate potential CAI dog’s predisposition to CAI’s. Altered behavioral image; improved opportunity of rehoming through association with CAI dogs.	Behavioral freedom.	Intrinsic value; being recognized as being of the same intrinsic value as non-shelter dogs; same duty of responsibility and care for them. Being given an equal opportunity to find a good home/to live a life worth living. Being recognized as a sentient being.
**OTHER CAI DOGS**	Welfare, health, wellbeing; safety; avoid overburdening due to more dogs being available for CAI.	Behavioral freedom; possibility to exit CAI if appropriate. Increased opportunity of matching the dog to the specific CAI due to the availability of more individuals to choose from. Less pressure to retain a CAI dog who seeks to avoid CAI interaction.	Being recognized as having intrinsic value. Being recognized as a sentient being.
**OTHER PROFESSIONALS IN SHELTERS (behaviorists, managers, operators, dog trainers, volunteers)**	Acceptable workload and working conditions. Possibility to minimize/be protected from compassion fatigue and moral distress. Less stress over resource limitations, due to cost savings associated with adoption of shelter dogs.	Managerial freedom; adequate training.	Adequate resources for sustainable and smart management (including adequate resources to allow dogs to retain and nurture a predisposition for CAI (if applicable) in the shelter environment).
**VETERINARIANS in shelters**	Acceptable workload and working conditions. Adequate payment for the extra responsibility. Possibility to minimize compassion fatigue and moral distress.	Managerial freedom; adequate training. Adequate resources and freedom to decide when/if to carry out tests to screen for possible CAI related risks.	Adequate resources for sustainable and smart management. Same societal/economic consideration as any other similar professional not working in a shelter.
**VETERINARINS/BEHAVIOURISTS** **involved in CAI**	Increased working opportunities. Adequate payment for their work.	Adequate training to deal with challenges associated with shelter dogs Possibility to decide when not to involve dogs from shelters in CAI.	Possibility to minimize the negative effects of the responsibility for the possible extra risks of involving shelter dogs vs. non-shelter dogs. Being given the same consideration/respect as that given to CAI veterinarians/behaviorists working with non-shelter dogs.
**OTHER PROFESSIONALS IN CAI (dog handlers, dog trainers, therapists, teachers, coaches)**	Increased working opportunities.	Adequate training to deal with challenges associated with shelter dogs. Possibility to decide when/when not to not involve dogs from shelters in CAI.	Same (very low!) risk of being harmed by participating in CAI with shelter dogs as that of people participating in CAI with non-shelter dogs. Being given the same consideration/respect as that given to CAI veterinarians/behaviorists working with non-shelter dogs.
**PATIENTS/CLIENTS OF CAI**	Increased health and safety. Increase in CAI dogs available to provide CAI.	Informed choice to participate in CAI with dogs from shelters.	Availability and affordability of interventions. Same (very low!) risk of being harmed by interaction in CAI with shelter dogs as that of people participating in CAI with non-shelter dogs.
**PUBLIC AUTHORITY**	Reduction in public action required in the management of shelter dog population.	Possibility to develop better management of public economic resources.	Reduction in public economic waste.
**SOCIETY**	Increased health and safety. Reduction in environmental impact of overcrowded shelters. Reintegration of dogs in society. Positive companion animal partnerships. Cost savings associated with adoption of shelter dogs.	Freedom to address social resources for other animals, human, or environmental issues.	Increased opportunity to offer CAI services for vulnerable people and for all of society.

## Data Availability

No new data were created or analyzed in this study.
